# Bridging the gap between basic science and clinical practice: The role of organizations in addressing clinician barriers

**DOI:** 10.1186/1748-5908-6-35

**Published:** 2011-04-04

**Authors:** Megan Beckett, Elaine Quiter, Gery Ryan, Claude Berrebi, Stephanie Taylor, Michelle Cho, Harold Pincus, Katherine Kahn

**Affiliations:** 1 RAND Health, Santa Monica, California, USA; 2 RAND Health-University of Pittsburgh Health Institute, Pittsburgh, Pennsylvania, USA; 3 Department of Psychiatry, Columbia University, New York, New York, USA; 4 Division of Quality and Safety, New York-Presbyterian Hospital, New York, New York, USA; 5 Department of Medicine, David Geffen School of Medicine at UCLA, Department of Medicine, Los Angeles, California, USA; 6 UCLA School of Public Health, Community Health Sciences, Los Angeles, California, USA; 7 Compass Lexecon, Oakland, CA, USA

## Abstract

**Background:**

New National Institutes of Health policies call for expansion of practice-based research to improve the clinical research enterprise and facilitate dissemination of evidence-based medicine.

**Objective:**

This paper describes organizational strategies that influence clinicians' decisions to participate in clinical research.

**Design:**

We reviewed the literature and interviewed over 200 clinicians and stakeholders.

**Results:**

The most common barriers to community clinician participation in clinical research relate to beliefs that clinical research is too burdensome and has little benefit for the participating clinician or patient. We identified a number of approaches healthcare organizations can use to encourage clinicians to participate in research, including an outreach campaign to promote the benefits of clinical research; selection of study topics of interest to clinicians; establishment and enforcement of a set of research principles valuing the clinician and patient; development of a transparent schedule of reimbursement for research tasks; provision of technological and technical assistance to practices as needed; and promotion of a sense of community among clinicians involved in practice-based research.

**Conclusions:**

Many types of existing healthcare organizations could provide the technical and intellectual assistance community clinicians need to participate in clinical research. Multiple approaches are possible.

## Background

The National Institutes of Health (NIH) and other policymakers have identified broader recruitment of community-based physicians and their patients into large-scale clinical studies as a priority for the national health research agenda [1-3]. Meeting this goal will require recruiting many clinicians who would not typically participate in clinical research [2-4]. The NIH [5] has called for practitioners to commit to stable, long-term participation. Thus, recruitment and training need to be tailored to meet this expectation. We and others have identified many barriers to long-term participation in clinical research by community physicians and other clinicians, including time constraints, insufficient staff and training, concern about negative impact on doctor-patient relationships, clinician discomfort with the informed consent process, and overly rigid eligibility requirements [6-11]. Interestingly, many of the factors practitioners identify as barriers to participation in clinical research also contribute to inefficiencies in clinical care (for example, rising costs, slow results, and fragmented infrastructure) [12]. Thus, identifying support services that improve the efficiency of medical practice as well as research and lead to reduced costs should also make it easier to recruit community practitioners for long-term research participation.

## Methods

We conducted a literature review and interviews with over 200 clinicians and stakeholders to identify factors that limit or enhance community clinician involvement in clinical research, and to examine the role that healthcare organizations might play in removing the barriers to involvement. Interviewees were identified through 'snowball sampling,' with efforts made to ensure a diverse sample with respect to demographics as well as knowledge and experience base. Snowball sampling is a technique for developing a research sample where existing study subjects recruit future subjects from among their acquaintances. Between September 2004 and September 2005, a total of 243 participants were interviewed, including 112 clinicians whose reports of barriers to participation in research informed this analysis (see Kahn et al., for more detailed description of the research methods [6]). The remaining participants with expertise in the conduct of clinical research in community settings contributed to our development of strategies for addressing clinicians' concerns.

After each interview, the interviewer(s) identified key themes and issues that arose during the interview and presented these at a weekly investigator debriefing meeting. This approach served to facilitate rapid sharing of new information and themes identified and to identify issues that should be further developed in upcoming interviews. Additionally, two or more investigators reviewed all transcripts within two weeks of the interview to identify key themes. This attention to detail resulted in a key issues content change between the early and the late interviews.

Kahn *et al. *described the barriers we identified for clinician participation in research within the context of their own community practices and proposed a number of innovations to remove them [6]. In the Results section of this article, we document concerns reported by clinicians that influence their decisions about whether or not to incorporate clinical research into the context of their community practices. We also present respondent reports of suggested strategies to overcome concerns. We organized this text around a model of clinical research participation decision-making selected and adapted by the research team based on our study results [13].

Recognizing the importance of partnerships and infrastructure in supporting complex activities, such as sustained research activities within the context of ongoing clinical practice, we then present strategies that healthcare organizations can use to address clinician concerns. These strategies should increase the likelihood that clinicians will choose -- in a sustained way -- to incorporate into their practices research that could inform clinical questions, methods, analyses, and clinical recommendations built upon their own patients. These strategies are derived from the research team's synthesis of clinician self-reports, literature review, and internal discussions. We probed informants and searched the literature to identify potential strategies. We then conducted further interviews of community clinicians and other key stakeholders for their assessments of these strategies and the roles that healthcare organizations might play. Where relevant, we applied potential strategies to specific barriers or issues identified through clinician self-report interviews.

The RAND institutional review board (IRB) reviewed these materials and procedures prior to the start of the interviews.

## Results

**Figure 1:**
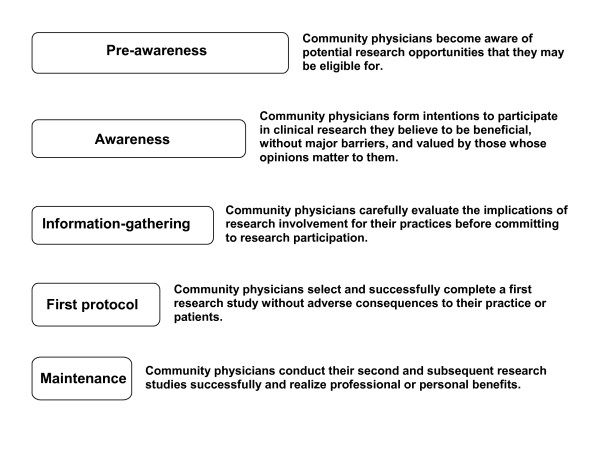
**A Decision model to participate in clinical research studies **[33].

The results of our initial interviews and literature review suggested that the decision-making processes of clinicians contemplating participation in clinical research are similar to the decision-making processes of people contemplating changes in health behaviors (Figure 1) [13]. We next organized the potential strategies for overcoming clinician concerns according to the stage of the research participation model most directly addressed by each strategy (see Table 1).

**Table 1:** Overcoming barriers to community clinician-participation in clinical research through organizational support

Model phase	Barriers to participation	Organizational solutions
Pre-Awareness	1. Community clinicians do not know about clinical research studies they may be eligible to participate in.	1. Conduct a multi-media outreach campaign to educate Clinicians about possible research opportunities. Identify and reach all potentially eligible Clinicians.
Awareness	1. Clinicians do not appreciate potential personal or professional benefits to clinical research. They feel research questions are not pertinent to their patients.	1. As part of an outreach campaign, craft messages that highlight benefits to Clinicians and patients, as well as the value of clinical research to the organization. When possible, provide relevant research studies with topics of interest to clinicians, with minimum exclusion criteria, or solicit clinician input on these areas and channel results to study sponsors.
	2. Clinicians believe that research tasks are too difficult to successfully implement in community practice.	2. As part of an outreach campaign, identify and address barriers to research participation that are especially problematic within clinicians' systems, and effectively communicate to clinicians how these barriers will be addressed.
	3. Clinicians do not believe that clinical research is valued by colleagues or patients.	3. Develop and articulate clinical research principles that value clinicians and patients and with a clear set of standards that clinicians and other research participants must adhere to.
Information-Gathering	1. Clinicians have insufficient information or ability to carefully evaluate the business, clinical, and resource implications of participating in research.	1. Provide informational and instrumental assistance pertinent to the business implications of participation. Explain how reimbursement is set for clinical research. Explain study protocol and training requirements. Review checklist for costs. Evaluate and check appropriateness of liability insurance.
	2. Clinicians face considerable uncertainty about levels of reimbursement they can expect and about future additional research opportunities they can expect.	2. Develop a reimbursement schedule for research tasks clinicians can peruse to assess impact of clinical research on practice revenue. Post planned future studies on a Web-based registry to enable planning for future research engagement.
First Protocol	1. Clinicians have insufficient time or resources to register and train for a first protocol. For example, they do not know how to confirm with insurer that liability covers research-related tasks, interact with IRB, etc.	1. Provide informational and instrumental assistance from selection of the first protocol through to its successful completion. Review management and fiscal aspects of all procedures/tasks with clinicians. Explain the study reimbursement schedule including reimbursements and insurance coverage. Clarify areas of uncertainty. Provide administrative and technical support as needed. Work with clinicians to complete IRB requirements--identify and guide interactions with IRBs. Ensure that all administrative requirements are met.
	2. Clinicians do not know which protocol will provide the best 'fit' with the practice and maximize likelihood of meeting recruitment goals, collecting quality data, and not disrupting patient care and administrative tasks.	2. Provide consultation on protocol selection, setting enrollment goals and study timelines, and on QA procedures.
	3. Clinicians and their staff are uncertain about how to most effectively adjust workflow to accommodate research tasks.	3. Provide technology and technical support, such as a personal computer, fax machine with encryption software, and telephone computer support to help practices that do not have the technology or expertise.
Maintenance	1. Clinicians suffer repeated financial losses from research involvement.	1. Set or advocate for transparent study reimbursement schedules that fairly compensate clinicians for their time and effort. Provide consultation and audits to clinicians throughout each study as needed.
	2. Clinicians fear loss of patients to specialists because of research-related referrals.	2. Establish ethical principles of research that emphasize that poaching will not be tolerated, and closely monitor all principles.
	3. Clinicians do not feel that they are valued for their intellectual and clinical contributions--that they are being used for their patients.	3. Encourage a sense of research community. Recognize clinicians for their contribution and expertise. Provide a confidential venue such as a website portal to register queries/complaints/concerns about current and future protocols, continue to solicit clinician opinion about future research topics.

### Pre-awareness

With their main responsibility being the clinical care of patients, many clinicians said that they were unaware of opportunities to engage in clinical research. Consequently, some clinicians said that they would consider participating in a clinical research study, but they did not know where to learn about such opportunities.

### Awareness

Simply knowing about clinical research opportunities is insufficient. When we asked community clinicians what factors shape their attitudes toward clinical research, we learned that even if they were informed of research opportunities, they would need to value clinical research and believe that it is potentially beneficial to their patients and practices to be willing to invest the time and energy necessary to evaluate whether a particular research project would make sense for them. Some clinicians mentioned the benefits of intellectual stimulation and personal satisfaction derived from being a part of clinical trials. As one clinician observed, 'being able to participate can sometimes be exciting because you hear about cutting-edge things. And that can be rewarding.'

Other clinicians emphasized that they would participate only if the study topic was pertinent to their interests or practices or if they were familiar with the treatment being considered. Some community clinicians stated that many research questions were irrelevant to their practices or that most studies have overly rigid eligibility requirements that would disqualify many of their patients [11,14,15]. Clinicians also reported they would be more likely to commit to clinical research if colleagues, patients, and healthcare organizations valued their participation. For example, one informant noted that clinicians might participate if 'their participation is seen as valuable' by patients, other clinicians, and the clinical research staff. Some clinicians expressed distrust or the belief that their patients distrust clinical research [16,17]. Community clinicians often expressed the concern that they or their patients might be exploited for research purposes, and that interest in their participation in research might not be based upon their potential intellectual and professional contributions.

### Information-gathering

If clinicians were aware of and interested in clinical research, they still need to evaluate its financial, liability, and resource implications carefully. Clinicians stressed that preservation of a viable clinical practice is of paramount concern. As an informant from a clinical research organization noted:

'People don't do this for the greater good. They don't have time, and they can't afford to do it. They do it because it's a reasonable business proposition that also blends with their medical interest. You really have to think about this as, am I going to be able to, at the end of the day, pay my staff, pay my rent, and bring home enough money to make this worth doing? This is an additional possible activity to get involved in, and it's got to make some economic sense.'

Deciding if and how incorporating clinical research might jeopardize or benefit the practice requires more time and expertise than reported as available for most clinicians who have not participated in a research study. Despite time constraints, prior to initiating research participation, clinicians said it is critical to make time to accurately estimate how clinical research is likely to affect patient load, patient flow, and other essential practice characteristics. Specifically, clinicians reported that they could benefit from help thinking through the 'business implications of participating in protocols, staffing needs associated with research activities, strategies for adjusting an office practice so that clinical research does not adversely affect patient flow, and identifying ways to assure high quality data'. Clinicians said they need detailed information about what types of research tasks they will be asked to conduct. Clinicians also reported they want to be fairly reimbursed for their research activities. Clinicians with research experience told us that reimbursement levels for pharmaceutical studies are break-even, and that those for NIH-sponsored research fall short of cost. Such observations have led to calls for greater equity in reimbursement across types of studies [18].

### Participating in a research study

If a clinician decides that a clinical research study makes sense for the practice, he or she may decide to participate. Community clinicians who were experienced researchers overwhelmingly emphasized several problems they encountered in their initial research experience. Frequently mentioned were burdens associated with IRB applications, report generation, and communication with the principal investigator, data-coordinating center, and regulatory agencies regarding protocol design, protocol changes, IRB changes, and data quality. Both individual providers and leaders of large provider or research organizations emphasized that the Health Insurance Portability and Accountability Act (HIPAA), in particular, fundamentally changed the way data were gathered and disclosed, mystified many providers, and was enough to keep them from participating in clinical trials. Several informants said that access to shared and experienced study coordinators or nurses can help new researchers avoid or minimize these kinds of problems, enhancing the probability that initial research experiences are successful. One research network representative noted that 'a lot of the work we can't necessarily turn over entirely to the practices and the practitioners because they just don't have the manpower.' By using a study coordinator, 'one investigator can do a lot of research because they're typically only involved in actually seeing the patient at the first and last visit. The study coordinator sees all the patients in between.'

Clinicians without prior research experience also cited the need for technological or technical support. Clinicians observed that some practices might need a fax machine with encryption software, a DSL line, or an extra computer.

### Maintenance

If a clinician's first research experience is successful -- *e.g.*, the research protocol is well integrated into practice workflow and the practice loses no (or minimal) revenue -- the clinician is likely to participate in subsequent studies. An unsuccessful research experience may discourage a clinician, particularly one who is inexperienced at practice-based research, from participating in a subsequent study. As one informant observed, 'The challenge is 90% of the 46,000 [providers who filled out a statement of Investigator (Form FDA 1572)] will never do the third trial. But they get into it thinking they were going to start a research business. They walk in and anoint their office nurse study coordinator for the day. They do two trials and they look at each other and say, boy this is a whole lot harder than we ever thought. We're not going to do this any more.' More experienced clinicians indicated they require less administrative and technical support. However, they said they could still benefit from assistance in setting and meeting enrollment goals so that recruitment efforts and research activities do not swamp their clinical responsibilities. They may also need some assistance with quality assurance checks and audits.

## Discussion

We identified a series of concerns voiced by clinicians about barriers to clinician participation in clinical research. Building upon the model described above to account for decision making about participation in clinical research and the research team's synthesis of clinician self-reports, literature review, and internal discussions, the research team developed specific strategies that healthcare organizations could use to address clinician concerns.

### Strategies for overcoming barriers to clinical engagement, by decision-making stage

The research team identified themes, drew from the literature, and probed with subsequent informants about potential strategies for overcoming barriers to clinical research in an iterative process. Below, we describe the resulting strategies, organized by stage of our conceptual decision-making model.

### Pre-awareness

Organizations can address a lack of awareness of research opportunities by carefully designing outreach campaigns [12,16,18,19] for community clinicians. Care should be taken to ensure that all potentially eligible clinicians are identified and reached through multiple venues, including professional journals and meetings, websites for professional practice organization, newsletters, emails, mailings, and word-of-mouth.

Practice-based research networks (PBRNs) have successfully raised awareness of community clinicians regarding participation in research studies by encouraging them to preferentially participate in studies consistent with their personal and professional areas of interest [19]. Furthermore, PBRNs preferentially recommend community practitioners' participation in studies with designs that demonstrate the feasibility of conducting research in community-based clinical practice.

### Awareness

Clinicians reported that they are more likely to commit to clinical research if their patients, colleagues, and healthcare organizations value their efforts. An outreach campaign could be crafted to emphasize the multiple ways that clinical research can benefit clinicians and their patients. It might emphasize the potential of research within clinical practice to (1) contribute to evidence-based care including better understanding of which interventions improve their patient outcomes, (2) serve as a sign that the practice employs state-of-the-art medicine, and (3) engender professional recognition with peers.

Outreach campaigns would also need to address *risks *-- perceived by patients and clinicians -- of engaging in clinical research. In particular, outreach campaigns could address concerns that clinical research participation is exploitative of the clinician or patient. To increase clinician confidence that they and their patients will be well-treated, organizations trying to recruit clinicians to practice-based research could, in consultation with clinician and patient representatives, adopt and enforce a set of clinical research principles emphasizing value for clinicians and patients as integral components of the research endeavor. These principles would be most effective if they are clearly and consistently articulated in all outreach campaigns and embodied in all aspects of the research process, including the management and administrative structures supporting the clinical research endeavor. Emphasizing patient and clinician participation in IRB committees might also be useful. Examples of research principles to explicitly guide community clinician-organization interactions include:

• Avoidance of community clinicians being unduly pressured to participate in clinical research.

• Restraint by clinicians of enrollment of so many patients that they compromise their clinical responsibilities.

• Specification in protocols of all data quality requirements and procedures.

• Assurance that mechanisms are in place to support that clinicians have rapid and effective feedback of their data quality (such as through an internet-based registry).

• Commitment by participating clinicians that patient-poaching by specialists will not be tolerated.

• Implementation of publicized research principles to assure clinicians and their patients that they are valued as an integral component of the research endeavor.

Finally, clinicians often expressed reluctance to engage in practice-based research if they believed the clinical research tasks would be onerous and difficult to incorporate into patient care. Healthcare organizations that seek to promote research among their own clinicians need to address these commonly perceived barriers -- such as time constraints and lack of staffing and training -- within their own systems and effectively communicate to clinicians how this will be accomplished.

An effective and comprehensive outreach campaign will likely require more space and time than most media can provide. An internet-based registry (such as the one described in Kahn et al. [6]) could feature a portal that provides detailed description of the features, benefits, and requirements of clinical research and that clinicians can access at their convenience. Table 2 lists strategies for using a registry to support community-based clinician participation in research. One outreach strategy that is likely to be even more effective than a media campaign or a web portal is academic detailing. Academic detailing involves a short (10-minute) visit or discussion by an opinion leader or specialized clinician with a physician, either one-on-one or with a small group of physicians working in the same practice. Academic detailing has been found to be an effective method of changing physician behavior (such as improving adherence to clinical guidelines) [20,21].

**Table 2:** An internet-based registry that targets all stages of the model

Model stage	
Pre-Awareness	The organization can advertise the Web-link in journals and other media/venues that community clinicians routinely use.
Awareness	A portal on the registry could outline the personal and professional benefits of clinical research and describe how clinical research can be incorporated into practice with an emphasis on ways that common barriers will be overcome. The registry could also summarize a set of clinical research principles adopted by the organization to emphasize that clinicians and patients will be treated with integrity. A portal with a chat room could allow interested clinicians to talk with clinicians currently involved in clinical research about their experiences confidentially.
Information-gathering	Each research study can be characterized on the Web in terms of: objectives; expected benefits to science, patients and clinicians, including reimbursement rates; potential risks; types of tasks; and resources required. A registry can also post current and upcoming protocols so that clinicians can assess the predictability of clinical research studies over time.
First protocol	Web-links to research training, including IRB-training and patient-consenting procedures as well as specifics for a particular protocol, can be included. The registry can provide confirmation of the receipt of data/specimens and feedback about data quality and problems to confidentially assist clinicians to improve. Electronic chat sessions within the registry can provide peer support and a sense of community.
Maintenance	The registry can provide confirmation of the receipt of data/specimens and feedback about data quality and problems to confidentially assist clinicians to improve. Electronic chat sessions within the registry can provide peer support and a sense of community.

### Information-gathering

To help clinicians and medical practices realistically think through the implications to their practices of clinical research and ways to adjust their office practice, organizations can provide informational and decision-making assistance. Such assistance may be most effective if provided by a highly credible (to the clinician) professional with several years' experience working with medical practices and clinical research.

To address concerns that the reimbursement for time spent in clinical research activities are too low, organizations can advocate for equitable reimbursement for clinicians or, if the organizations themselves or a vendor are setting reimbursement rates, they can develop a reimbursement schedule for specific research tasks. These schedules can be posted in an easy-to-understand format on a website or in print. Transparent and easy-to-use information on reimbursement rates for a research protocol can be useful for clinicians who are selecting their first or subsequent protocol. Organizations can also identify procedures and visits that are covered by health insurance on behalf of practices. Organizations can help clinicians calculate whether staff time is covered, and whether this coverage is sufficient, as well as develop review checklists for costs. Clinicians may also need help determining whether their liability coverage is appropriate for engaging in research.

To provide clinicians with realistic expectations about what they would be expected to do prior to committing to participate in their first clinical research study, the internet-based registry can list all current and upcoming protocols along with associated tasks, clinician experience and training requirements, exclusionary criteria, and reimbursement schedule. Electronic chat sessions for clinicians interested in clinical research could contribute to a sense of community and provide a way for clinicians in the information-gathering stage to obtain advice from clinicians currently engaged in clinical research.

### Participating in a research study

Although this did not emerge from our discussions with clinicians, healthcare organizations can facilitate the completion of a clinician's first study at every step. Organizations can provide informational and instrumental assistance, ranging from selection of a protocol that is a good fit for practice resources and limitations, to thoroughly understanding and meeting protocol and training requirements. If a clinician fails to meet a requirement or does not understand the need for a specific supporting document, assistance can be given to overcome such obstacles. Links to training sites and other information can also be made accessible on the internet-based registry. In addition, the registry can help clinicians and organizations track completion of eligibility requirements. Organizations can also help community clinicians set enrollment goals and intervene if the clinician falls short of these goals. For example, a worksheet might guide efforts to ensure that processes and staff are in place to meet study timelines and data quality assurance processes; help identify certified laboratories and pharmacies; and coordinate referrals for multi-clinician studies meet all administrative requirements. A healthcare organization can also ensure that clinicians are provided with the technical and technological support, along with user support, needed to incorporate clinical research more easily into their practices.

Two other ways organizations can support clinicians during their initial research experiences are by rigorously applying and enforcing principles of research throughout the protocol and by promoting a sense of community. Venues like internet chat rooms that encourage clinicians to express themselves might help foster this sense of community, if such resources are accessed.

### Maintenance

The need to foster a sense of community among clinicians who participate in research continues after the first research experience, even if subsequent research projects require less administrative and technical support. Organizations, in conjunction with other clinician researchers, can foster a sense of community and responsibility for the research enterprise among all practicing clinicians involved in clinical research. For example, clinicians can be recognized at local and national meetings for their participation in research and encouraged to give presentations describing their research involvement and their particular studies and outcomes. Organizations can solicit clinician input on study topics and protocol development and channel this information to study sponsors. In turn, this could improve protocol applicability in community settings and communicate to clinicians that they are valued for their clinical expertise. A confidential venue (such as through a registry portal) could be provided for clinicians' 'voices' to be heard and for clinicians to register complaints or concerns about current and future protocols and ensure that protocols are feasible in their community settings.

### Strategies and organizational limitations

Not all of the strategies outlined above are suitable for all types of organizations that might be interested in supporting clinician involvement in clinical research, and may not apply to all clinical settings. Table 3 summarizes the levels of centralization and organizational size that might be required to successfully implement each strategy, consistent with the opinions expressed by our interviewees. A highly centralized healthcare organization is one that employs and directly influences the way its clinicians practice (such as a staff-model health maintenance organization, or HMO). A less centralized organization might be a specialty society that represents its members but exerts no control over them. The size of an organization can indicate its capacity to run multiple studies over time, reflecting the number of clinicians and patients involved in the organization and how much of the business can be devoted to research. Some examples of large clinical care organizations are academic health centers (AHCs) and large multispecialty clinics with a substantial research focus such as Kaiser-Permanente, Palo Alto Medical Clinic, Fallon Clinic, Marshfield Clinic, and the Mayo Clinic.

**Table 3:** Appropriateness of approach to support community clinician involvement in clinical research by organizational characteristic, size, and locus of control

	Organizational management level (high versus low)	Size of organization (small versus large)	Locus of control
Outreach campaign	Low or high	Small or large	Centralized or decentralized
Provide (or collect and channel to study sponsors) appropriate research questions	High	Large	Centralized
Articulate ethical research principles	High	Small or large	Centralized
Registry	High	Large	Centralized
Provide informational and administrative support	Low or high	Small or large	Centralized or decentralized
Develop transparent reimbursement rates	High	Small or large	Centralized
Provide technological and technical assistance	Low or high	Small or large	Centralized or decentralized
Promote a sense of community	Low or high	Small or large	Centralized or decentralized

Some of these approaches require a large, highly centralized organization (or highly centralized coalitions of smaller organizations), whereas others will require significant additional resources. Developing a registry and designing relevant research questions are likely to be the most challenging steps to fostering research: the cost and effort required to develop a registry and promote relevant research questions are best born by higher levels of organizational management that have control over sufficient resources to make them happen. Conversely, development and dissemination of research principles and the setting of reimbursement rates need to be completed at a relatively high level within an organization and require a high degree of centralization, although neither of these latter two approaches is particularly dependent upon the size of the organization.

Regardless of the size of the organization, senior leadership is critical to changing an organization's culture from one where research is not widely accepted to one where staff and management support and actively engage in clinical research. Senior leadership has been essential in changing physician behaviors, such as the adoption and use of new chronic disease management strategies within organizations [22]. Senior leadership is able to affect physician change by assessing challenges and opportunities within the organization, setting performance expectations for all staff and clinicians, aligning structures and functions, and engaging others within the organization [23]. In other words, senior management needs to be involved in all stages of the effort to change an organization's culture and its physician performance so that clinical research is valued and practiced.

## Summary

In this article, we outline a model for thinking about how organizations can influence community clinicians' decision-making about becoming or remaining involved in clinical research. We recommend a variety of approaches organizations can take to encourage clinicians to become involved in clinical research. The suggested approaches could be used singly or in combination by organizations to augment individual clinician efforts and address the realities of clinical practice today. Some of the solutions we propose, such as internet portals and chat rooms, have been tried and have not generally been effective. We believe that more intensive types of clinician outreach, in particular, academic detailing, will be necessary to modify physician behavior with respect to involvement in clinical research. Web portals and chat rooms, in contrast, are likely to be most effective in transmitting additional information to clinicians who are interested or involved in clinical research and are seeking more specific information (*e.g.*, maintenance phase).

Healthcare organizations are a promising vehicle through which support services can be delivered to large numbers of clinicians [24]. Accordingly, healthcare organizations currently leading quality improvement efforts that target clinical care inefficiencies are excellent candidates for modifying systems to support clinical research. Some healthcare organizations, such as AHCs [25], other academic research organizations [26], PBRNs [17,27], clinical research organizations [28], and clinical trials networks [29], have a strong tradition of promoting clinical research among affiliated clinicians. Policymakers are now recommending the formation of coalitions between traditional research-oriented organizations (like AHCs) and other healthcare organizations (such as multispecialty groups, HMOs, community hospitals, and ambulatory medical practices) with the goal of improving desired outcomes (*e.g.*, provision of evidence-based care, improved quality of care scores) [2,3]. NIH's current focus on community clinicians [2] may heighten the motivation for organizations to support this group's efforts to participate in research.

A limitation to our conceptual model of clinician decision-making for the adoption of clinical research is that it is based on what clinicians report would influence their decision to participate or not. We did not observe their behavior. Self-report by clinicians does not always track behavior. Thus, the proposed approaches to improving clinician involvement in the research endeavor should be subjected to empirical study.

Our review of the literature on clinical trial design reveals numerous recommendations aimed at enhancing participation in practice-based research that are consistent with the model we have developed. These recommendations include outreach to clinicians (and patients) about the importance of clinical research, distribution of research tasks among clinic staff, standardization of data collection procedures, and dissemination of technology to alert clinicians automatically about upcoming clinical trials [10,14,19,30,31]. Such approaches have thus far had a modest impact [17]. More effort is needed to increase incentives and remove barriers to improve community clinician recruitment and long-term involvement in clinical research [8,32].

## Competing interests

The authors declare that they have no competing interests.

## Authors' contributions

MB, GR, and KK designed the study and drafted the manuscript. EQ, CB, ST, MC, and HP guided study design and read and revised the manuscript. All authors read and approved the final manuscript.
